
JOURNAL INFORMATION
==============================
NLM Title Abbreviation: Wirtschaftsdienst
Journal ID: Wirtschaftsdienst
NLM Journal ID: 101086612

Wirtschaftsdienst (Hamburg, Germany : 1949)

ISSN: 0043-6275

ARTICLE INFORMATION
==============================
PMCID: PMC7964515
PMID: 33746301
DOI: 10.1007/s10273-021-2868-7
Article ID: 2868
Article version: 1
Subjects: Zeitgespräch

Ein Investitionsprogramm zur Krisenbewältigung

Clemens Marius MClemens@diw.de
1
Fratzscher Marcel MFratzscher@diw.de
2
Michelsen Claus cmichelsen@diw.de
1
1 grid.8465.f 0000 0001 1931 3152 Konjunkturpolitik, Deutsches Institut für Wirtschaftsforschung, Mohrenstr. 58, 10117 Berlin, Deutschland
2 grid.8465.f 0000 0001 1931 3152 Deutsches Institut für Wirtschaftsforschung, Mohrenstr. 58, 10117 Berlin, Deutschland
Electronic publication date: 2021 Mar 17
Print publication date: 2021
Volume: 101
Issue: 3
First page: 168
Last page: 171
Copyright: © Der/die Autor:in(nen) 2021
License: Open Access: Dieser Artikel wird unter der Creative Commons Namensnennung 4.0 International Lizenz veröffentlicht (https://creativecommons.org/licenses/by/4.0/deed.de).
Open Access wird durch die ZBW — Leibniz-Informationszentrum Wirtschaft gefördert.
License URL: https://creativecommons.org/licenses/by/4.0/

issue-copyright-statement: © The Author(s) 2021

==============================

In den USA ist eine intensive Debatte über die Größe und die Wirkung finanzpolitischer Impulse entbrannt. Ein Paket von rund 1,9 Billionen US-$ hat die Biden-Administration angekündigt, zusätzlich zu dem Ende 2020 beschlossenen Paket von 900 Mrd. US-$, um die Folgen der Corona-Krise aufzufangen. Larry Summers und Oliver Blanchard befürchten, dass diese massive Ausgabensteigerung die Inflation erhöhen könnte. Das beschlossene Ausgabenprogramm ist insgesamt viermal größer als das der Finanzkrise und etwa drei bis fünf mal so groß wie die aktuelle Produktionslücke. Paul Krugman hingegen argumentiert, in einer Gesundheitskrise seien zunächst alle Mittel zu ergreifen, um diese zu beenden. Zudem wäre der Zusammenhang zwischen Arbeitslosigkeit und Inflation strukturell bedingt schwächer – ein Fiskalimpuls der die Produktionslücke schließt oder gar überkompensiert würde demnach nicht zu massiver Inflation führen.[1] In der EU dagegen ist die Produktionslücke größer, der fiskalische Impuls aber deutlich kleiner als in den USA. Klar ist, dass die Corona-Krise weltweit massive wirtschaftliche Schäden verursacht. In Deutschland betrug die Produktionslücke 2020 nach herkömmlicher Schätzung in etwa -5,5 % oder gut 180 Mrd. Euro (Gemeinschaftsdiagnose, 2020). 2021 dürfte sie noch bei bis zu -4 % bzw. 130 Mrd. Euro liegen. Zwar gehen die meisten Ökonom:innen davon aus, dass die Pandemie im Jahresverlauf bewältigt werden kann – sicher scheint dies aber keinesfalls. Zu unklar ist, wie erfolgreich die globalen Impfkampagnen sein werden, ob Mutanten des Coronavirus zu immer neuen Infektionswellen führen und welchen Flurschaden die Pandemie in der Unternehmenslandschaft oder auf den Arbeitsmärkten hinterlassen wird.

Die öffentlichen Haushalte müssen derzeit nicht fürchten, dass die Schulden- bzw. die Zinslasten kurzfristig zu Problemen führen werden. Im Gegenteil: derzeit kann der Bundesfinanzminister Geld damit verdienen, wenn er Kredite aufnimmt. Die Zinsen für deutsche Wertpapiere sind negativ und dürften so schnell auch nicht steigen. In einer solchen Situation scheint es sinnvoll, eher zu viel als zu wenig Geld in die Hand zu nehmen, um die Krise und deren wirtschaftlichen Folgen zu bekämpfen. Hinzu kommt, dass es in Deutschland – unabhängig von der aktuellen Krise – einen erheblichen Bedarf für öffentliche Investitionen gibt. Verschiedene Berechnungen aus Zeiten vor der Corona-Pandemie zeigen, dass bis 2030 zwischen 300 Mrd. und 450 Mrd. Euro für den Erhalt und den Ausbau der Infrastruktur in Deutschland benötigt werden (Bardt et al., 2019; Krebs und Scheffel, 2018; Barišić, Krebs und Scheffel, 2018; Fratzscher, 2015). Zwar wurden in den letzten Jahren die öffentlichen Investitionsetats hochgefahren, auch das Konjunkturprogramm aus dem Sommer 2020 enthält entsprechende Mehrausgaben. Die hier benannte Größenordnung ist allerdings noch nicht in den Haushaltsplänen vorgesehen, sodass weiterhin eine erhebliche Lücke besteht (Belitz et al., 2020a) (vgl. Tabelle 1). Dieser Investitionsbedarf dürfte in der Krise kaum kleiner geworden sein. Es spricht sogar vieles dafür, dass jetzt ein günstiger Zeitpunkt für ein umfassendes Modernisierungsprogramm ist, das die konjunkturelle Entwicklung stabilisiert, das Wachstumspotenzial steigert und den Wirtschaftsstandort für die kommenden Herausforderungen wettbewerbsfähig macht (Belitz et al., 2020b).

## Crowding-in oder -out durch öffentliche Investitionen?

Eine wichtige Funktion öffentlicher Investitionen ist es, private Investitionen zu ermöglichen, die vor allem für die langfristige Transformation der Wirtschaft – in Bezug auf Klimaschutz, Digitalisierung und Innovation – essenziell sind. Grundsätzlich kann eine Erhöhung öffentlicher Investitionen mit verschiedenen, auch gegensätzlich wirkenden makroökonomischen Effekten einhergehen. Zunächst induzieren zusätzliche öffentliche Ausgaben rein technisch einen Anstieg des Bruttoinlandsprodukts

1 https://paulkrugman.substack.com/p/stagflation-revisited.

Tabelle 1

**Investitionsbedarfe und Ausgaben bis 2030**

in Mrd. Euro

| | Bildung | Wohnungsbau | Digitalisierung | Klima und Verkehr | Gesundheit | Volumen[a] |
|---|---|---|---|---|---|---|
| Investitionsbedarf[b] | 100 | 50 | 60 | 120 | 0 | 330 |
| - Maßnahmen der großen Koalition[c] | 17 | 3 | 18 | 21 | 0 | 59 |
| = Investitionsbedarf abzüglich Beschlüsse | 83 | 47 | 42 | 99 | 0 | 271 |
| + Gesundheitssektor | | | | | 10 | 10 |
| Investitionsbedarf vor Konjunkturpaket | 83 | 47 | 42 | 99 | 10 | 281 |
| - Konjunkturpaket 2020[d] | 5 | 0 | 16 | 30 | 10 | 61 |
| darunter | | | | | | |
| Zukunftspaket[e] | 2 | | 16 | 30 | | 48 |
| Ganztagsschulen und Kitas | 3 | | | | | 3 |
| Gesundheitspaket | | | | | 10 | 10 |
| offener Investitionsbedarf[f] | 78 | 47 | 26 | 69 | 0 | 220 |

[a] Volumen berechnet sich als Gesamtbedarf bis 2030. [b] Investitionsbedarf nach Krebs und Scheffel (2017) bis 2030 fortgeschrieben. [c] Berücksichtigung der Maßnahmen aus dem Koalitionsvertrag für jeweils unterschiedliche Dauer: Digitalpakt Schule bis 2024, Breitbandausbau bis 2025, Strukturstärkungsgesetz: Kohleregionen bis 2038, Ausbau des Schienennetzes bis 2030, Fonds zur Förderung von KI bis 2025, Kommunalinvestitionförderungsgesetz bis 2022, EKF-Ausgaben (ohne Bahn) bis 2023. [d] Investive und konsumtive Maßnahmen des Konjunkturprogramms in den Bereichen Bildung, Wohnungsbau, Digitalisierung, Klimaschutz, ÖPNV und kommunaler Infrastruktur. [e] Investive und konsumtive Maßnahmen in den Bereichen Bildung, Wohnungsbau, Digitalisierung, Klimaschutz, ÖPNV und kommunaler Infrastruktur. [f] ohne zusätzliche Bedarfe im Zusammenhang mit dauerhaften Auswirkungen der COVID-19-Pandemie.

Quelle: Eigene Zusammenstellung und Berechnungen.

(BIP) in gleicher Höhe. Darüber hinaus wird die kurzfristige Nachfrage nach privaten Investitionen, Konsum oder Importen beeinflusst (Abiad, Furceri und Topalova, 2016). Schließlich erhöhen öffentliche Investitionen den Kapitalstock und damit das Produktionspotenzial (Krebs und Scheffel, 2017; Ramey, 2020). Dabei können gegensätzlich wirkende Effekte auftreten, die die private Konsum- und Investitionsnachfrage entweder stimulieren (Crowding-in) oder verdrängen (Crowding-out).

Crowding-in bedeutet ein Anschieben der privaten Nachfrage durch staatliche Investitionen. Ein Grund hierfür ist, dass durch staatliche Investitionen private Güter vermehrt nachgefragt und diese effizienter produziert und bereitgestellt werden können. Z. B. vereinfacht und forciert eine staatlich finanzierte Ausweitung des Straßenverkehrsnetzes den Transport und Handel von Gütern und Dienstleistungen. Private Unternehmen erwarten somit Effizienzgewinne in Produktionsprozessen und höhere Profite, sodass privatwirtschaftliche Anreize zu verstärkter Investitionstätigkeit bestehen. Firmen, die mit diesen zukünftigen Produktivitätsgewinnen sicher planen können, werden bereits in der Gegenwart investieren. Sind diese Produktivitätsgewinne unsicher, weil etwa der Staat seine Investitionstätigkeit nach Kassenlage ausrichtet, werden Unternehmen dementsprechend ihre eigene Investitionstätigkeit reduzieren. Die Erhöhung öffentlicher Investitionen führt folglich im theoretischen Kontext zu einer Erhöhung der Grenzproduktivität des privaten Kapitals und einer effektiven Erhöhung der Nachfrage nach privaten Gütern. Beim Crowding-out-Effekt verdrängen öffentliche Investitionen die privatwirtschaftliche Nachfrage. In der Literatur werden Crowding-out-Effekte meist daraus abgeleitet, dass der Staat zur Finanzierung von öffentlichen Investitionen neue Staatsanleihen begibt. Die Konsequenz der dadurch gestiegenen Kreditnachfrage sind höhere Zinsen und somit steigende Kapitalkosten. Dies verschlechtert wiederum die Finanzierungsbedingungen der Unternehmen, weshalb Investitionen aufgeschoben werden oder sogar vollständig ausbleiben könnten. In Phasen von anhaltenden Niedrigzinsen dürfte dieser Kanal allerdings deutlich abgeschwächt sein. Aber der Staat fragt bei einer Erhöhung der öffentlichen Investitionen auch Arbeitskräfte nach, die er den privaten Firmen folglich potenziell entzieht. Dies dürfte wiederum vor dem Hintergrund bereits auftretender demografisch bedingter Arbeitskräfteengpässe ein zunehmend wichtiger Faktor werden. Zudem kann der Staat durch seine zusätzliche Nachfrage auch das allgemeine Preisniveau erhöhen, wodurch Unternehmensgewinne und Haushaltseinkommen in realer Rechnung geringer ausfallen können.

## Investitionen in Krisen mit hoher Rendite

Welcher Effekt unter welchen Bedingungen überwiegt, ist eine empirische Frage, der in einer aktuellen Studie auf Grundlage einer Vielzahl von Schätzmodellen nachgegangen wurde (Belitz et al., 2020a).[2] Die Analysen richten sich dabei auf unterschiedliche Investitionsarten, Zeiträume sowie Branchenunterschiede. Zum einen werden Investitionen nach enger (VGR)-Abgrenzung, d. h. Bau-, Ausrüstungs- und sonstige Investitionen berücksichtigt. Zum anderen werden auch Investitionen im weiteren Sinne erfasst, d. h. auch Humanpotenzialinvestitionen (Dullien et al., 2020), die in der Studie anhand der Ausgaben in den Bereichen Bildung, Erziehung und Gesundheit gemessen werden, die aus rein VGR-rechnerischer Perspektive dem Staatskonsum zugeordnet werden. Zudem werden die

2 Unter anderem werden strukturelle VAR-Modelle, ein Proxy-VAR mit einer narrativen Investitionszeitreihe aus den Finanzberichten von 1970 bis 2019, Local-Projection-Schätzungen unter anderem mit Prognosefehlern der Gemeinschaftsdiagnose sowie ein bayesianisch geschätztes Fiscal-DSGE-Modell für Deutschland herangezogen. Die Schätzergebnisse werden zudem durch diverse Robustheitsteste validiert.

Abbildung 1
**Durchschnittlicher Crowding-in Effekt öffentlicher Investitionen in kurzer und mittlerer Frist**

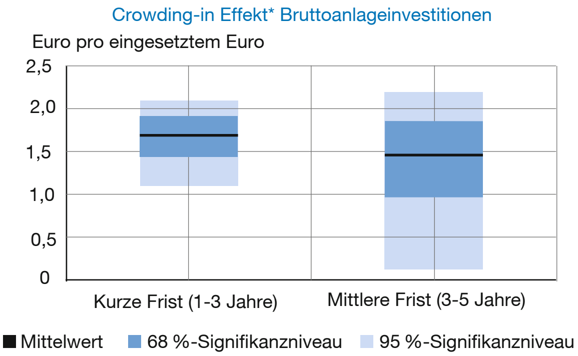

* im Durchschnitt über alle Monate.

Quelle: Belitz et al. (2020a).

Abbildung 2
**Zustandsabhängiger Crowding-in Effekt öffentlicher Investitionen**

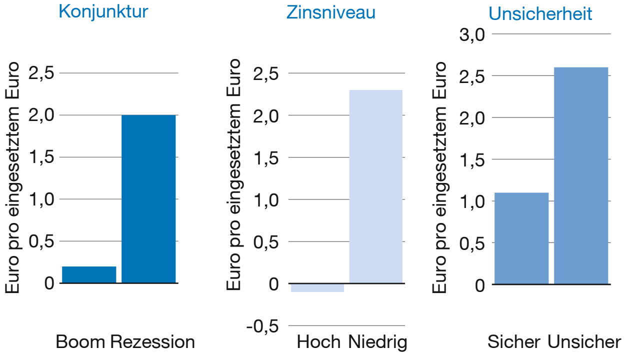

Quelle: Belitz et al. (2020a).

Effekte auch im Hinblick auf unterschiedliche ökonomische Zustände, wie die konjunkturelle Lage, die aktuelle Zinspolitik oder auch die wirtschaftliche Unsicherheit, untersucht. So können die Effekte beispielsweise in einer konjunkturellen Überauslastung anders wirken als in einer Phase der Unterauslastung oder Rezession. Es zeigt sich, dass öffentliche Investitionen im Durchschnitt über alle Modelle, Fristigkeiten, Investitionsarten und ökonomischen Zustände die privaten Investitionen erhöhen. So führen öffentliche Investitionen in Höhe von einem Euro zu einer zusätzlichen privaten Investitionstätigkeit von etwa 1,50 Euro (vgl. Abbildung 1). Das BIP steigt dabei in ähnlicher Größenordnung.

Auch lässt sich für einige Investitionsarten, wie z. B. F&E- und Humanpotenzialinvestitionen sowie zum Teil auch Bauinvestitionen ein Produktivitätseffekt nachweisen, der erst in der mittleren Frist zu steigenden privaten Investitionen führt und der besonders stark ist, wenn der Investitionsbedarf hoch ist. Ein kurzfristiger, konjunkturell stimulierender Effekt geht von öffentlichen Bau- und Ausrüstungsinvestitionen auf die privaten Bauinvestitionen aus. Denn öffentliche Bau- und Ausrüstungsinvestitionen sorgen dafür, dass private Unternehmen in Ausrüstungen investieren, unter anderem allein schon um öffentliche Bauaufträge ausführen zu können. Diese kurzfristigen Effekte verschwinden allerdings im Zeitablauf. Längerfristiger können aber einige auch selbst direkt von der staatlichen Infrastruktur profitieren wollen und den Standort verlagern bzw. sich neu ansiedeln. Darüber hinaus zeigt sich die Relevanz ökonomischer Zustände (vgl. Abbildung 2). Der Crowding-in-Effekt ist in den allermeisten Fällen positiv. Gerade in Phasen der wirtschaftlichen Unterauslastung ist er besonders hoch. Dies spricht dafür, dass öffentliche Investitionen in Zeiten, in denen die Kapazitäten gering ausgelastet sind, große positive Effekte auf die gesamtwirtschaftliche Nachfrage entfalten. Ähnlich hohe Crowding-in-Effekte lassen sich auch für Zeiträume nachweisen, in denen das Zinsniveau niedrig ist. Umgekehrt überwiegt in Hochzinsphasen der Crowding-out-Effekt. Auch wirtschaftliche Unsicherheit ist ein Zustand, in dem öffentliche Investitionstätigkeit stabilisierender wirkt und dadurch private Investitionstätigkeit stärker anregt als in sicheren Zeiten. Im aktuellen Umfeld der Corona-Krise (Rezession, niedriges Zinsniveau, hohe Unsicherheit) sollten deshalb deutlich höhere Stabilisierungs- und Wachstumseffekte aus öffentlichen Investitionen entstehen als in Phasen der Normalauslastung.

## Investitions- und Wachstumsprogramm für Deutschland

Die Ergebnisse legen nahe, dass sich ein Investitionsprogramm in der aktuellen wirtschaftlichen Lage doppelt auszahlen würde. Die Wirtschaft ist in der Unterauslastung, die Unsicherheit über die zukünftige Entwicklung ist hoch und die Zinsen äußerst niedrig. Gleichzeitig besteht Konsens über die Notwendigkeit umfangreicher staatlicher Investitionen für Dekarbonisierung, Digitalisierung, Forschung und Entwicklung, Bildung aber auch die gebaute Infrastruktur. So sieht auch die Bundesregierung im Juni 2020 beschlossenen Konjunkturprogramm ein „Zukunftspaket" mit öffentliche Investitionen und Investitionszuschüssen in diesen Bereichen in Höhe von rund 43 Mrd. Euro bis 2024 vor. Den Modellschätzungen zufolge könnte 1 Euro dieser öffentlichen Ausgaben kumuliert bis 2024 etwa 1,60 Euro zusätzliches BIP mit sich bringen (vgl. Abbildung 3). Dadurch läge das reale BIP bis 2024 jahresdurchschnittlich um etwa 0,4 % höher als ohne das Programm. Insbesondere im Niedrigzinsumfeld, durch das die Finan-

Abbildung 3

**Private Investitionen und BIP infolge der öffentlichen Investitionen (Bruttoanlageinvestitionen, Investitionszuschüsse, Humanpotenzialausgaben) des Konjunkturprogramms vom Juli 2020**

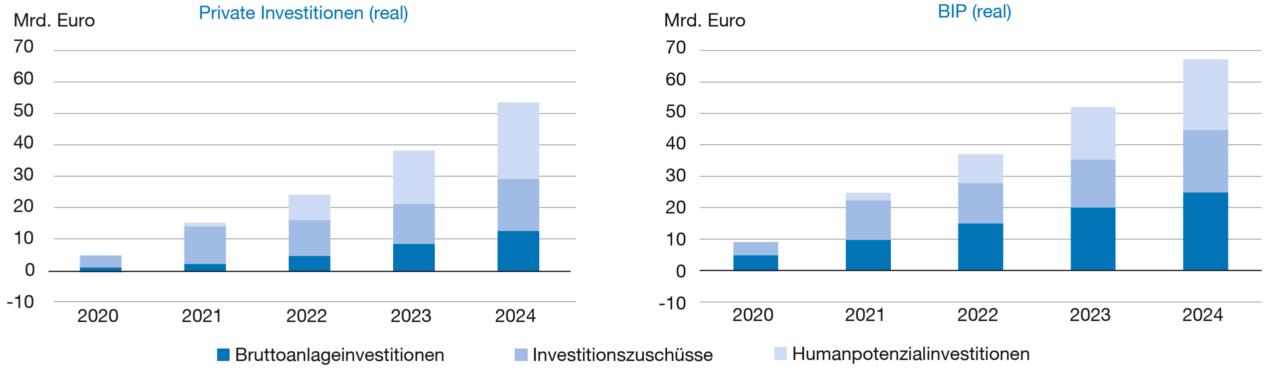

Quelle: Belitz et al. (2020a).

zierungskosten bei der Ausgabe von Staatsschuldtiteln wegfallen bzw. sogar zu realen Finanzierungsgewinnen werden können, sollte das zusätzliche Wachstum auch einen spürbaren Beitrag bei der mittelfristigen Rückführung der öffentlichen Verschuldung liefern.

Doch auch wenn die Bundesregierung seit 2018 mit verschiedenen investiven Maßnahmen in Höhe von zusammengenommen rund 110 Mrd. Euro die Modernisierung der Volkswirtschaft fördert, existiert bis 2030 weiterhin ein öffentlicher Investitionsbedarf von rund 220 Mrd. Euro. Insbesondere in den Bereichen Bildung, Klimaschutz und Wohnbau sowie im Bereich der Infrastruktur auf kommunaler Ebene ist der Bedarf weiterhin groß. Entsprechende Schwerpunkte in den Haushalten von Bund, Ländern und Kommunen zu setzen, erscheint auch deshalb sinnvoll, weil damit die demografisch bedingten Verluste des Potenzialwachstums teilweise aufgefangen werden könnten. Mit dem Anstieg des Produktionspotenzials werden die öffentlichen und privaten Kapazitäten ausgeweitet bzw. Produktionsprozesse effizienter, z.B. durch digitalisierte Schulen und öffentliche Verwaltungen oder auch eine bessere Kinderbetreuung. Auch kurz- und mittelfristige Nachfragengpässe, die durch den wirksamen Fiskalimpuls zu steigenden Preisen führen könnten, können dadurch abgemildert werden, dass ein Ausgabenprogramm stärker langfristig auf investive Bedarfe und die Modernisierung der Volkswirtschaft ausgerichtet ist und so nicht nur die private Nachfrage einmalig oder nur über wenige Jahre stimuliert wird, sondern auch die volkswirtschaftlichen Produktionskapazitäten permanent erhöht werden. Dies bedeutet, dass es langfristig keinen Zielkonflikt zwischen einem Abbau der Staatsschuldenquote und höheren öffentlichen Ausgaben für Investitionen gibt, wenn diese Investitionen das Potenzialwachstum erhöhen, dadurch die Steuereinnahmen stärken und die Sozialsysteme entlasten. Gegenwärtig hat Deutschland einen großen Bedarf an solchen Investitionen. Dies ist ein zentrales Resultat vorliegender Studien, das für die gegenwärtige wirtschaftspolitische Debatte in Deutschland von großer Bedeutung ist.

Dr. Marius Clemens ist wissenschaftlicher Mitarbeiter am Deutschen Institut für Wirtschaftsforschung (DIW Berlin).

Prof. Dr. Marcel Fratzscher ist Hochschullehrer an der Humboldt-Universität zu Berlin und Präsident des DIW Berlin.

Dr. Claus Michelsen leitet die Abteilung Konjunkturpolitik am DIW Berlin.


Literatur

Abiad A Furceri D Topalova P The Macroeconomic Effects of Public Investment: Evidence from Advanced Economies Journal of Macroeconomics 2016 50 C 224 240 10.1016/j.jmacro.2016.07.005
Bardt, H., S. Dullien, M. Hüther und K. Rietzler (2019), Eine solide Finanzpolitik: Investitionen ermöglichen, IMK Report, 152, November, Institut für Makroökonomie und Konjunkturforschung.
Barišić M Krebs T Scheffel M Eine Investitionsagenda für Deutschland Wirtschaftsdienst 2018 98 3 179 185 10.1007/s10273-018-2260-4
Belitz, H., M. Clemens, S. Gebauer und C. Michelsen (2020a), Öffentliche Investitionen als Triebkraft privatwirtschaftlicher Investitionstätigkeit, DIW Politikberatung kompakt, 158.
Belitz H Clemens M Fratzscher M Gronig M Kemfert C Kritikos A S Michelsen C Neuhoff K Rieht M Spieß C K Mit Investitionen und Innovationen aus der Corona-Krise DIW Wochenbericht 2020 87 24 442 451
Dullien, S., M. Hüther, T. Krebs, B. Praetorius und C. K. Spieß (2020), Weiter Denken: ein nachhaltiges Investitionsprogramm als tragende Säule einer gesamtwirtschaftlichen Stabilisierungspolitik, DIW Politikberatung kompakt, 151.
Fratzscher, M. (2015), Increasing Investment in Germany: Report Prepared by the Expert Commission on Behalf of the Federal Minister for Economic Affairs and Energy, Expertenkommission Stärkung von Investitionen in Deutschland.
Gemeinschaftsdiagnose (2020), Erholung verliert an Fahrt — Wirtschaft und Politik weiter im Zeichen der Pandemie, 2.
Krebs, Tom und M. Scheffel (2016), Quantifizierung der gesamtwirtschaftlichen und fiskalischen Effekte ausgewählter Infrastruktur- und Bildungsinvestitionen in Deutschland, Working Papers, 16–13, University of Mannheim, Department of Economics.
Krebs T Scheffel M Lohnende Investitionen Perspektiven der Wirtschaftspolitik 2017 18 3 245 262 10.1515/pwp-2017-0015
Ramey, V. A. (2020), The Macroeconomic Consequences of Infrastructure Investment, NBER Working Paper, 27625, National Bureau of Economic Research.
